# The supernatant of *Lactiplantibacillus plantarum* 25 is more effective than extracellular vesicles in alleviating ulcerative colitis and improving intestinal barrier function

**DOI:** 10.3389/fmicb.2025.1742486

**Published:** 2026-01-16

**Authors:** Shuang Gong, Xin Li, Qiong Zhang, Rui Wang, Ruixia Zeng, Yibo Zhang

**Affiliations:** 1 School of Basic Medical Sciences, Jinzhou Medical University, Jinzhou, Liaoning, China; 2 School of Stomatology, Shenyang Medical College, Shenyang, Liaoning, China; 3 Shenyang Key Laboratory of Prevention and Treatment of Systemic Important Diseases Associated with Oral Diseases, Shenyang, Liaoning, China; 4 Department of Human Anatomy, School of Basic Medical Sciences, Jinzhou Medical University, Jinzhou, Liaoning, China; 5 Department of Pathogenic Biology, School of Basic Medical Sciences, Jinzhou Medical University, Jinzhou, Liaoning, China; 6 Collaborative Innovation Center Zoonosis Prevention and Treatment of Jinzhou Medical University, Jinzhou, Liaoning, China; 7 Liaoning Province Key Laboratory of Human Phenome Research, Jinzhou Medical University, Jinzhou, Liaoning, China

**Keywords:** extracellular vesicles, intestinal microbiota, *Lactiplantibacillus plantarum*, tight junction protein, ulcerative colitis

## Abstract

**Introduction:**

*Lactiplantibacillus plantarum* (*L. plantarum*) has been reported to attenuate ulcerative colitis (UC) and restore intestinal barrier integrity. However, it remains unclear whether culture supernatant or extracellular vesicles (EVs) are more effective.

**Methods:**

UC was induced in mice to compare the effects of *L. plantarum* 25 (LP25) supernatant and EVs on disease severity, survival, and tight junction protein expression. Gut microbiota and metabolism were analyzed by 16S rRNA sequencing and untargeted metabolomics. *In vitro*, LPS-stimulated Caco-2 cells and a Caco-2/RAW 264.7 co-culture model were used to evaluate barrier integrity, immune responses, and TLR4/NF-κB pathway activation.

**Results:**

Compared with EVs, LP25 supernatant significantly improved survival, alleviated disease severity, preserved tight junction protein expression, modulated gut microbiota, enhanced intestinal functional protein expression, and inhibited macrophage TLR4/NF-κB activation.

**Discussion:**

LP25 supernatant exerts superior protective effects compared with EVs in alleviating UC and maintaining intestinal barrier function, highlighting its potential as a functional component for dietary interventions targeting inflammatory bowel diseases.

## 1 Introduction

Ulcerative colitis (UC) is a chronic inflammatory bowel disease (IBD) characterized by complex interactions among genetic susceptibility, environmental influences, intestinal microbiota and immune dysregulation (Clarke and Chintanaboina, 2019). Although current therapeutic strategies, including immunosuppressants and anti-inflammatory agents, can alleviate symptoms to some extent, their efficacy remains limited and is often accompanied by adverse effects (Ungaro et al., 2017). Even in acute UC, where spontaneous remission may occur, persistent disruption of the intestinal barrier and microbial imbalance continues to drive disease progression (Huang et al., 2022). Therefore, identifying novel therapeutic strategies that effectively restore intestinal homeostasis has become an urgent priority.

Probiotics have increasingly been recognized as promising candidates for UC management, primarily by modulating gut microbiota, enhancing barrier integrity, and regulating of host immune responses (Zhao and Jiang, 2021). Among these probiotics, *Lactiplantibacillus plantarum* has demonstrated remarkable efficacy in improving UC symptoms and repairing the intestinal epithelial barrier (Gu et al., 2023a). Its protective mechanisms involve cytokine-mediated immunomodulation, upregulation of TJ proteins and reshaping of gut microbial composition (Tanaka et al., 2020), collectively contributing to reduced inflammation and enhanced mucosal healing.

Disruption of the intestinal barrier represents a hallmark of UC pathogenesis (Liu et al., 2024). TJ proteins, including ZO-1, occludin and claudins, are essential components for maintaining epithelial integrity (Wells et al., 2017; Turner, 2009). Loss of these proteins impairs mucosal defense, permitting harmful microbes and macromolecules to penetrate the mucosa and exacerbate inflammation (Kobayashi et al., 2020; Torres et al., 2020; Dokladny et al., 2016). Therefore, reinforcing the intestinal barrier is considered a critical therapeutic approach for UC.

Previous studies have shown that specific *L. plantarum* strains or their extracellular vesicles (EVs) exert protective effects on the gut. For instance, EVs derived from *L. plantarum* UJS001 were reported to promote M2 macrophage polarization and modulate cytokine secretion, thereby alleviating intestinal inflammation (Ganapathy et al., 2022). Similarly, *L. plantarum* SC-5 mitigated dextran sulfate sodium (DSS)-induced colitis in mice by suppressing NF-κB and MAPK signaling, enhancing TJ proteins expression, and restoring microbial homeostasis (Chen et al., 2024). These findings suggest that different *L. plantarum*-derived components may exert distinct protective mechanisms.

Gut microbial dysbiosis represents another hallmark of UC (Shi et al., 2023). Alterations in microbiota composition can exacerbate inflammation through endotoxin overproduction and activation of pro-inflammatory pathways (Franzosa et al., 2019; Wang L. B. et al., 2022). Probiotics, including *L. plantarum*, have been shown to inhibit pathogenic bacteria, promote short-chain fatty acid (SCFA) production, and restore microbial diversity, thereby contributing to intestinal homeostasis (Wang X. et al., 2022; Zhou et al., 2018; Carpino et al., 2020). These findings highlights the therapeutic potential of probiotics and their derivatives in the management of inflammatory bowel diseases.

Our previous study demonstrated that EVs derived from LP25 suppress inflammatory responses and promote macrophage polarization toward an anti-inflammatory phenotype (Li et al., 2024). However, whether the supernatant of LP25 exerts superior effects compared with its EVs remains unclear. In this study, we directly compared the therapeutic efficacy of LP25-derived EVs and supernatant in a murine model of acute UC. We found that the supernatant conferred more pronounced protection by improving survival, restoring barrier integrity, modulating gut microbiota and inhibiting activation of TLR4/NF-κB signaling pathway. These findings provide novel insights into the potential application of *L. plantarum* supernatant as a cost-effective and promising therapeutic option for UC.

## 2 Materials and methods

### 2.1 Bacterium, supernatant and extracellular vesicles preparation

*Lactiplantibacillus plantarum* 25 (*L. plantarum* 25, LP25) LP25 was isolated from traditionally fermented dairy product “Nai Doufu” collected in Inner Mongolia, China, and has been deposited in the China General Microbiological Culture Collection Center (CGMCC No. 31750).

The strain LP25 was cultured in De Man-Rogosa-Sharpe (MRS) medium (Hope Bio-Technology Co., Ltd., Qingdao, China) at 37 °C for 72 h, the bacterial culture was then centrifuged at 8,000 × g for 15 min at 4 °C, and the resulting supernatants were filtered through a 0.22 μm membrane (Millipore, United States) to obtain the *L. plantarum* supernatant. LP25-derived extracellular vesicles (LP25 EVs) were isolated from the supernatant using ultracentrifugation as described previously (Gong et al., 2024).

### 2.2 Mouse model with DSS-induced colitis

Six-week-old male C57BL/6 mice (18 ± 2 g) were purchased from Changsheng Biotechnology Co. (Liaoning, China). The mice were housed under controlled environmental conditions (temperature, 23 ± 1 °C; relative humidity, 50 ± 5%; 12 h light/dark cycle). The animals had free access to autoclaved drinking water and a standard pelleted diet. The mice were acclimated under standard laboratory conditions for 1 week before experiments.

Colitis was induced by administering 3% (w/v) dextran sulfate sodium (DSS; MW 40,000; Macklin, China) in autoclaved drinking water for 7 consecutive days (*n* = 6). The DSS solution was freshly prepared every day. To compare the therapeutic efficacy of different LP25-derived components, mice were randomly divided into four groups: control, LP25 bacterial suspension (B-LP25, 10^9^ CFU/mL), LP25 extracellular vesicles (LP25 EVs, 2 mg/kg), and LP25 culture supernatant (LP25, 250 mg/kg). All treatments were derived from the same LP25 culture to ensure biological consistency. The development of colitis was monitored daily by recording body weight changes and calculating the disease activity index (DAI). On day 13, colon tissues and fecal samples collected from all mice for subsequent analyses.

A secondary UC mouse model was established, after which mice were administered LP25 EVs by intraperitoneal injection at either 1 mg/kg (L-EVs) or 2 mg/kg (H-EVs), or LP25 supernatant by oral gavage at 125 mg/kg (L-LP25) or 250 mg/kg (H-LP25).

### 2.3 Evaluation of the severity of colitis

Body weight changes, disease activity index (DAI) scores, and colon length were assessed according to the DAI evaluation criteria described previously (Manicassamy and Manoharan, 2014) and summarized in Supplementary Table S1. Upon completion of the experiment, all mice were euthanized via cervical dislocation under isoflurane anesthesia. The length of the colon was subsequently measured and recorded.

### 2.4 Cell culture

Caco-2 cells were seeded in 6-well plates at a density of 2 × 10^6^ cells per well. When cell confluence reached 80–90%, the complete culture medium was replaced with serum-free high-glucose DMEM (Gibco, United States). Cells were treated with lipopolysaccharide (LPS, *Escherichia coli* serotype O111: B4, Sigma-Aldrich, United States) at a final concentration of 10 μg/mL, either alone or in combination with LP25 supernatant or extracellular vesicles (EVs). For co-treatments, cells received either low-concentration LP25 supernatant (L-LP25, 25 μg/mL) or low-concentration EVs (L-EVs, 120 μg/mL), or high-concentration LP25 supernatant (H-LP25, 250 μg/mL) or high-concentration EVs (H-EVs, 240 μg/mL). All treatments were performed for 24 h under standard culture conditions (37 °C, 5% CO_2_).

### 2.5 Quantitative real-time PCR

Total RNA was extracted using Trizol reagent (Vazyme, R401-01, Nanjing, China) according to the manufacturer’s instructions. RNA concentration and purity were determined using a UV spectrophotometer (HITACHI, Japan). Complementary DNA (cDNA) was synthesized using the ABScript Neo RT Master Mix kit (ABclonal, RK20433, Wuhan, China). Quantitative polymerase chain reaction (qRT-PCR) was performed using Genious 2X SYBR Green Fast qPCR Mix (ABclonal, RK21203, Wuhan, China) on a Gentier 96 Real-Time PCR System (Gentier 96, Tianlong, China). Relative gene expression levels were calculated using the 2^−ΔΔCt^ method with normalization to *β-actin* as the internal reference gene. The primer sequences used for qPCR are listed in Supplementary Table S2.

### 2.6 Cell viability assay

Cell viability was measured by Cell Counting Kit-8 (APExBIO, K1018, United States) according to the manufacturer’s instructions. Caco-2 cells seeded into in 96-well plates at a density of 5,000 cells per well and allowed to adhere overnight before treatment. Caco-2 cells were cultured to 80–90% confluence, the original medium was replaced and the cells were treated with LP25 supernatants at concentrations of 0.25, 2.5, 25, and 250 μg/mL or EVs at concentrations of 120, 240, 480, and 960 μg/mL. The mixtures were gently agitated, and wells containing only complete medium and untreated cells served as controls. Each concentration was tested in triplicate. After 24 h of incubation, the medium was removed, the cells were washed once with PBS, and fresh complete medium containing 10% CCK-8 was added. The plates were incubated at 37 °C for 2 h and absorbance was measured at 450 nm using a microplate reader (ALLSHENG, Hangzhou, China).

### 2.7 Western blot

Tissue and cell samples were lysed in NP-40 lysis buffer (Solarbio, Beijing, China) containing 1 mM phenylmethylsulfonyl fluoride (PMSF). Tissue homogenates were prepared using a glass homogenizer on ice, and all lysates were incubated for 30 min on ice with periodic mixing. The supernatants were collected after centrifugation at 12,000 × g for 10 min at 4 °C. Protein concentrations were determined using the BCA assay (Beyotime, China). Equal amounts of protein (20 μg per lane) were separated by 10–12% SDS-PAGE and transferred onto polyvinylidene fluoride (PVDF) membranes (Millipore, United States). Membranes were blocked with 5% non-fat milk in PBST for 2 h at room temperature and incubated overnight at 4 °C with rabbit primary antibodies against anti-ZO-1 (1: 1000), anti-Claudin-1 (1: 1000), anti-Occludin (1: 1000), anti-α-SMA (1: 500) (Affinity Biosciences, Cincinnati, United States), anti-Villin (1: 2000) (Proteintech, Chicago, United States), anti-intestinal alkaline phosphatase (IAP) (1: 5000) (Abcam, UK), anti-CD86 (1: 1000), anti-Arg-1 (1: 1000), anti-TLR4 (1: 500) (Affinity Biosciences, Cincinnati, United States), anti-NF-κB p65 (1: 2000) (Zenbio, Chengdu, China), anti-phospho-NF-κB p65 (1: 1000) (Abmart, Shanghai, China), anti- IκB-α (1: 5000), anti-phospho-IκB-α (1:2000) (Zenbio, Chengdu, China), and GAPDH (1:10000) (Affinity Biosciences, Cincinnati, United States). After washing with PBST, the membranes were incubated with secondary goat anti-rabbit IgG (1:8000) (Affinity Biosciences, Cincinnati, United States) at room temperature for 2 h. Finally, Protein bands were visualized using an enhanced chemiluminescence imaging system (CLiNX, Shanghai, China), and band intensities were quantified using ImageJ software (NIH, United States).

### 2.8 Histological analysis

For hematoxylin and eosin (H&E) staining, colon tissues were fixed in 4% paraformaldehyde at 4 °C for at least 24 h. The fixed tissues were then trimmed to appropriate sizes and rinsed under running tap water for 4–6 h. Subsequently, the samples were sequentially dehydrated in 70, 80, 95, and 100% ethanol, followed by clearing in xylene. Paraffin-embedded tissues were sectioned into 5 μm-thick slices using a microtome, deparaffinized in xylene, rehydrated through graded ethanol, and stained with H&E. The stained sections were then mounted with neutral resin and observed under a light microscope.

### 2.9 Immunofluorescence staining

Frozen colon tissue sections were equilibrated at room temperature for 40 min, washed three times with PBS for 5 min each, permeabilized with 0.3% Triton X-100 for 20 min, and blocked with 1% bovine serum albumin (BSA) for 2 h. The sections were then incubated overnight at 4 °C with primary antibodies against ZO-1 (1:200), occludin (1:100), and claudin-1 (1:100). After washing three times with PBS (10 min each), the sections were incubated with Alexa Fluor 488-labeled goat anti-rabbit IgG secondary antibody (1:500; Abmart, Shanghai, China) for 2 h at room temperature in the dark. Finally, the sections were mounted using antifade mounting medium containing DAPI (Solarbio, S2110, Beijing, China), and images were captured under a fluorescence microscope (Leica, Germany).

### 2.10 Microbiota 16S rDNA gene sequencing

Stool samples from each group were retrieved from −80 °C storage. Total genomic DNA was extracted from stool samples, and its concentration and purity were assessed using agarose gel electrophoresis and spectrophotometric analysis. The PCR amplicons were then quantified using fluorescence-based methods, and sequencing libraries were constructed by pooling the samples in proportion to the required sequencing depth. Sequencing was subsequently carried out on the NovaSeq 6000 platform (Illumina, United States). All 16S rRNA gene sequencing and bioinformatic analyses were performed by Baiqu Biotechnology Co., Ltd. (Shanghai, China).

### 2.11 Transwell co-culture system

An *in vitro* intestinal model was established using a co-culture system of Caco-2 and RAW 264.7 cells. To mimic inflammatory bowel disease (IBD) conditions, Caco-2 cells (2 × 10^5^ cells per well) were seeded onto the apical side of six-well Transwell insert plates (0.4 μm pore size; polyethylene terephthalate; 14112-D, LABSELECT, China) and allowed to differentiate for 21 days, with medium changes every 2 days. RAW 264.7 macrophages (2 × 10^6^ cells per well) were seeded into the basolateral chamber of the Transwell plates. Lipopolysaccharide (LPS, 1 μg/mL) was added to the basolateral compartment to induce an inflammatory response. LP25 supernatant was applied to the apical chamber at low (L-LP25, 25 μg/mL) or high (H-LP25, 250 μg/mL) concentrations and incubated for 18 h.

### 2.12 Untargeted metabolomics analysis of LP25 supernatant

A bacterial suspension of LP25 (10^9^ CFU/mL) was inoculated into 200 mL of MRS broth, while uninoculated MRS broth served as the control. The cultures were incubated at 37 °C under agitation at 200 rpm for 72 h. After incubation, the cultures were centrifuged at 4 °C and 4,000 rpm for 15 min, and the resulting supernatant was collected. The supernatant was transferred into a dialysis bag and concentrated at 4 °C using PEG-12000. The concentrated culture supernatant was analyzed for protein content using the BCA assay, filtered through a 0.45 μm membrane, aliquoted, and transported on dry ice to Shanghai Applied Protein Technology Co., Ltd. (Shanghai, China) for untargeted metabolomic profiling.

Based on the metabolomic data, differential analyses were conducted on all identified metabolites, excluding unknown compounds. Metabolites with a fold change (FC) > 1.5 or < 0.67 and *p* < 0.05 (*Student’s t*-test) were considered significantly altered. Visualizations were generated as volcano plots using the Microbiome Analyst platform.^1^

For KEGG pathway annotation and enrichment analyses, the top 50 differential metabolites in both positive and negative ionization modes were ranked by fold change. Metabolites with variable importance in projection (VIP) > 1 and *p* < 0.05 were defined as significantly altered and used for KEGG pathway annotation. The results were visualized as bubble plots using the same platform. Pathways with a rich factor > 0.15 were further screened, and within these pathways, metabolites with a fold change > 10 were identified as key differential metabolites.

### 2.13 Statistical analysis

All statistical analyses were performed using GraphPad Prism version 10.0 (GraphPad Software, San Diego, CA, United States). Data were expressed as the mean ± standard deviation (SD) from three independent experiments performed in triplicate. For comparisons between two groups, two-tailed *Student’s t*-tests were conducted. For multiple-group comparisons, one-way or two-way analysis of variance (ANOVA) was used as appropriate. A *p*-value < 0.05 was considered statistically significant.

For 16S rDNA sequencing data, the Kruskal-Wallis rank-sum test and Wilcoxon rank-sum test were employed to assess differences among groups. Differential taxa were identified using a significance threshold of *p* < 0.05.

## 3 Results

### 3.1 LP25 supernatant significantly improves physiological indicators in acute UC mice

**Figure 1:**
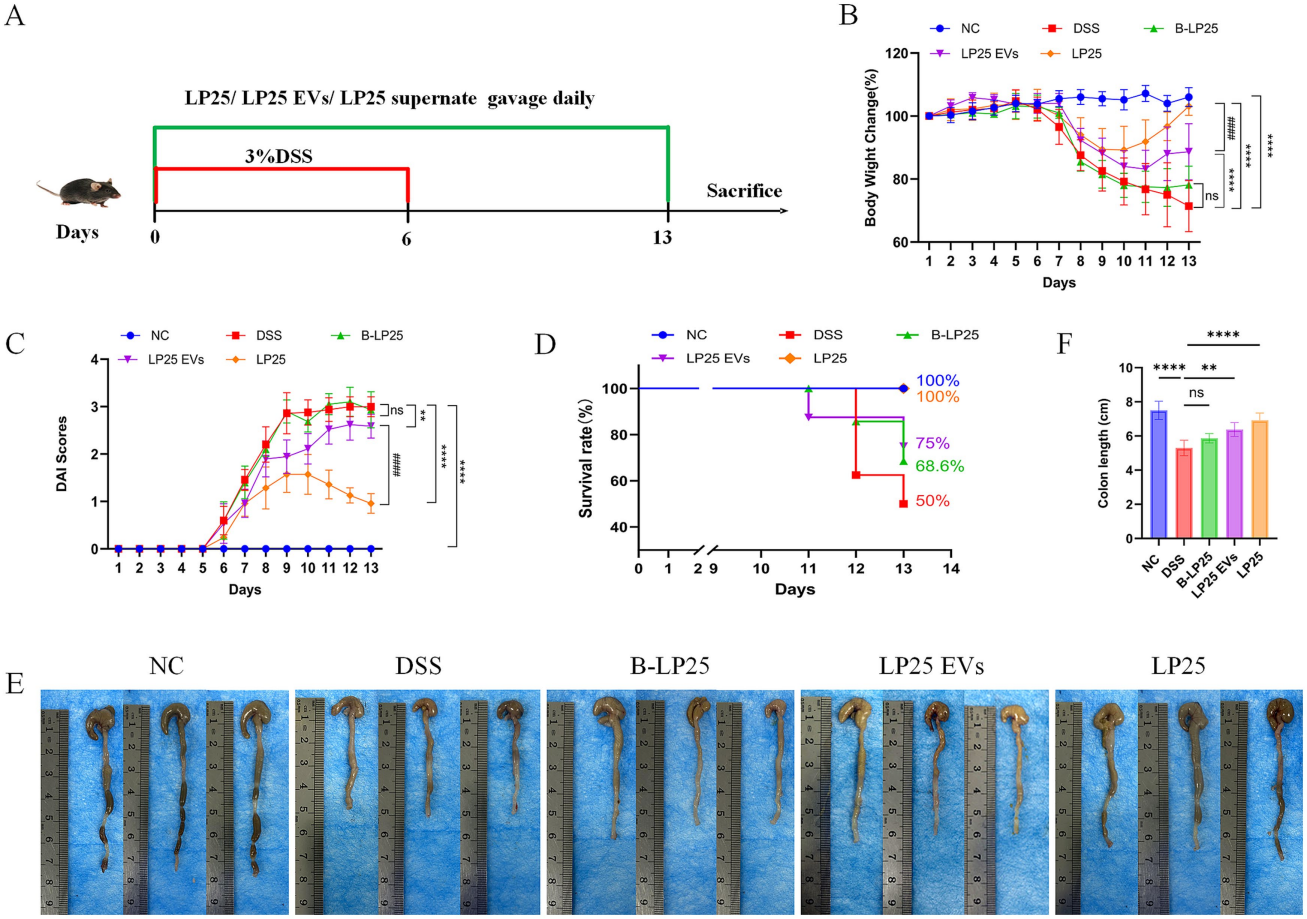
Effects of LP25 bacterial suspension, LP25-derived extracellular vesicles (EVs), and LP25 supernatant on body weight, disease activity index (DAI), survival rate, and colon length in DSS-induced acute ulcerative colitis (UC) mice. **(A)** Schematic illustration of the experimental design, showing induction of acute UC with 3% DSS and the oral administration schedule of LP25 preparations. **(B)** Changes in body weight among groups (LP25: 10^9^ CFU/mL; LP25 EVs: 2 mg/kg; LP25 supernatant: 250 mg/kg). **(C)** DAI scores. **(D)** Survival rates. **(E,F)** Representative images and quantitative comparison of colon lengths among groups. Data are expressed as mean ± SD (*n* = 6). ***p < 0.001, ****p < 0.0001 vs. DSS group; ns: not significant. The DSS group denotes mice receiving 3% DSS only.

Compared with the DSS group, the B-LP25 group showed no significant improvement in disease-related parameters. In contrast, both the LP25 supernatant and LP25 EVs groups exhibited significant improvements in physiological indicators. Specifically, the LP25 supernatant group showed increased body weight (Figure 1B), reduced DAI scores (Figure 1C), enhanced survival rates (Figure 1D), and elongated colon length (Figures 1E,F). Notably, these beneficial effects were more pronounced in the LP25 supernatant group than in the LP25 EVs group. The experimental design, including the induction of acute UC with 3% DSS and the oral administration schedule of LP25 preparations, is illustrated in Figure 1A.

### 3.2 LP25 supernatant more effectively promotes TJ proteins expression and alleviates UC-associated tissue injury

**Figure 2:**
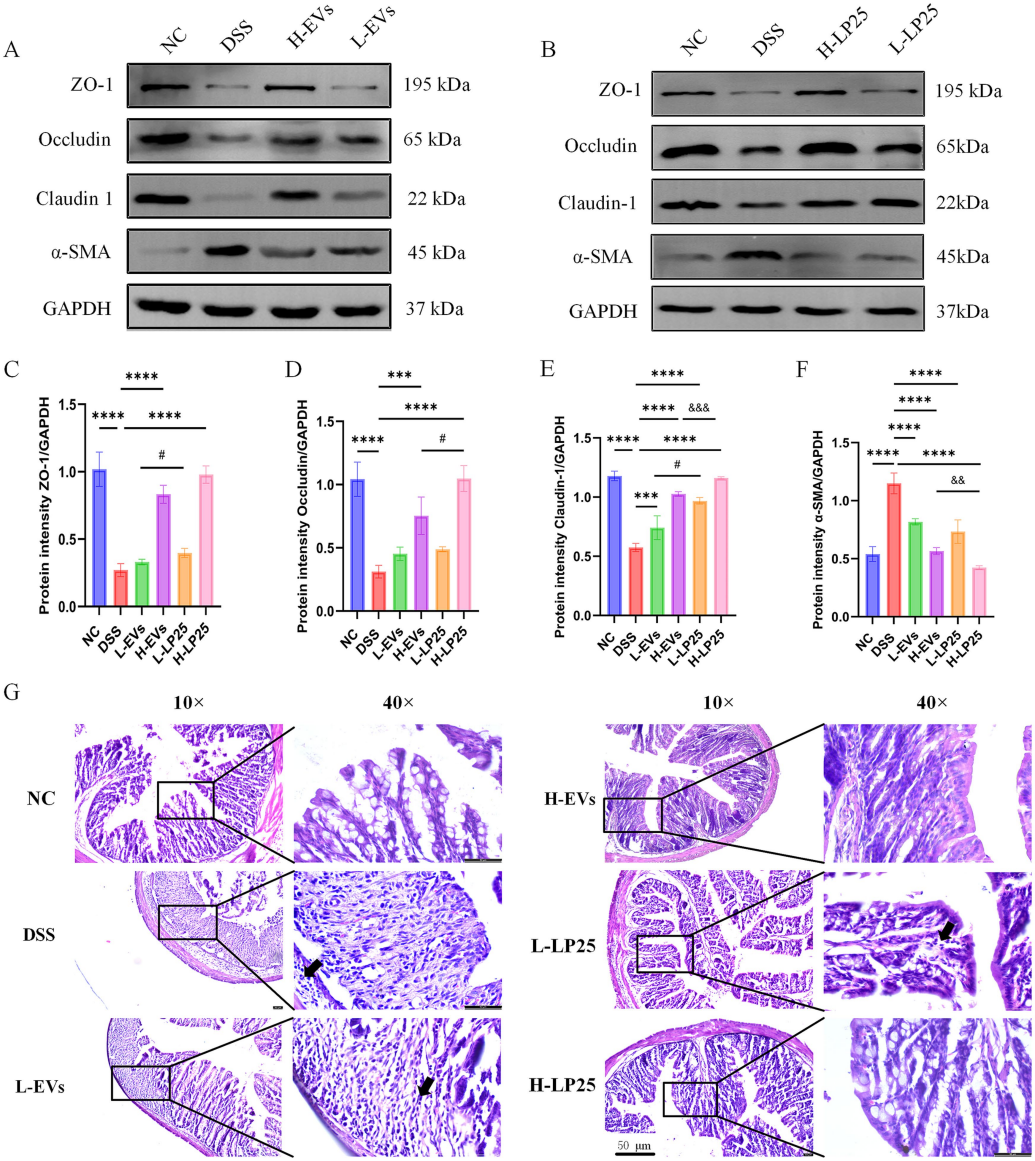
Effects of LP25-derived extracellular vesicles and LP25 supernatant on the intestinal mucosal barrier in acute ulcerative colitis mice. **(A,B)** Western blot analysis of barrier-associated proteins ZO-1, Occludin, Claudin-1, and α-SMA in colonic tissues from mice treated with 3% DSS alone, low-dose LP25 EVs (1 mg/kg), high-dose LP25 EVs (2 mg/kg), low-dose LP25 supernatant (125 mg/kg), and high-dose LP25 supernatant (250 mg/kg). **(C–F)** Quantitative densitometric analysis of the respective protein bands. **(G)** Representative hematoxylin and eosin (H&E) staining of colonic tissues from each group (100×, scale bar = 50 μm; magnified views at 400×). Black arrows indicate inflammatory cell infiltration. Data are presented as mean ± SD (*n* = 3). ***p < 0.001, ****p < 0.0001 indicate significant differences compared to the model group; ^#^*p* < 0.05, significant difference between low-dose LP25 EVs and low-dose LP25 supernatant groups; ^&&^*p* < 0.01, ^&&&^*p* < 0.001, significant differences between high-dose LP25 EVs and high-dose LP25 supernatant groups.

Both LP25-derived EVs and concentrated supernatant enhanced the expression of ZO-1, occludin and claudin-1 while reducing α-SMA levels; however, the supernatant exerted a significantly greater therapeutic effect than EVs (Figures 2A–F).

H&E staining showed normal histological architecture in the NC group as expected, whereas DSS treatment induced marked epithelial damage, inflammatory cell infiltration and goblet cell depletion. In mice treated with the LP25 supernatant, inflammatory infiltration was markedly reduced, goblet cell density increased, and crypt architecture was largely restored. In contrast, the EVs-treated group exhibited partial improvement with relatively preserved glandular structures and reduced inflammation, but substantial lesions remained (Figure 2G).

**Figure 3:**
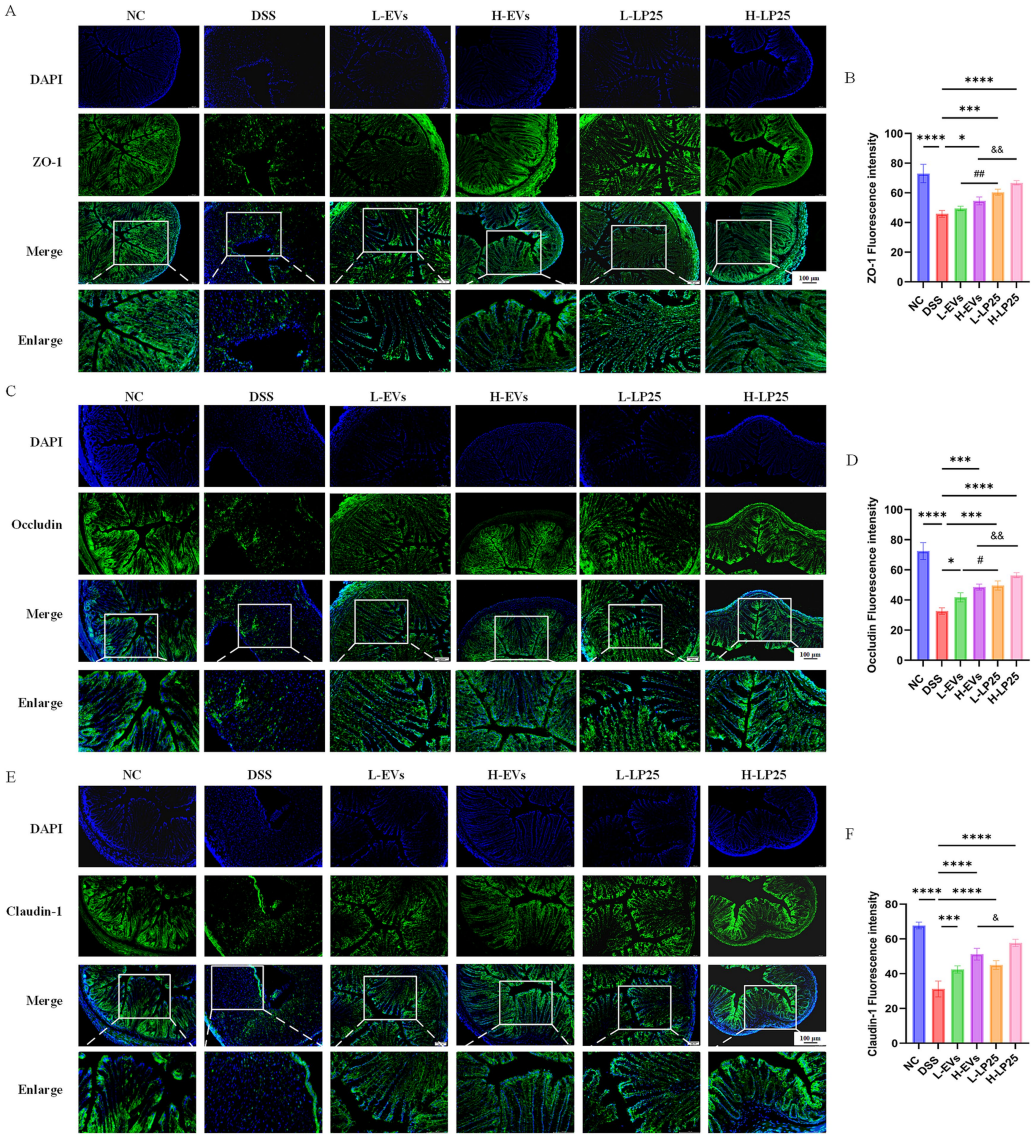
Effects of LP25-derived extracellular vesicles and LP25 supernatant on intestinal mucosal barrier integrity in DSS-induced acute ulcerative colitis mice. **(A–F)** Immunofluorescence staining of colonic tissues showing tight junction proteins ZO-1, Occludin, and Claudin-1 in each treatment group (magnification 10×; scale bar = 100 μm; insets show magnified images at 20×), along with corresponding fluorescence intensity quantification. Data are presented as mean ± SD (*n* = 3). *p < 0.05, ***p < 0.001, ****p < 0.0001 indicate significant differences compared to the model group; ^#^*p* < 0.05, ^##^*p* < 0.01 indicate significant differences between the low-dose LP25 EVs group and the low-dose LP25 supernatant group; ^&^*p* < 0.05, ^&&^*p* < 0.01 indicate significant differences between the high-dose LP25 EVs group and the high-dose LP25 supernatant group.

Consistently, immunofluorescence analysis revealed that the supernatant markedly increased ZO-1, occludin and claudin-1 expression compared with the DSS group, as evidenced by stronger fluorescence signals. Although EVs also improved TJ proteins levels relative to DSS, their effects were less robust than those of the supernatant (Figures 3A–F). Given the superior efficacy of LP25 supernatant in both *in vivo* and *in vitro* experiments, we subsequently investigated its influence on gut microbiota composition and explored potential bioactive components through metabolomic analysis.

### 3.3 LP25 supernatant modulates gut microbiota composition in UC mice

To investigate the effects of LP25 supernatant on intestinal microbial composition, 16S rDNA sequencing was performed on fecal samples from all groups.

**Figure 4:**
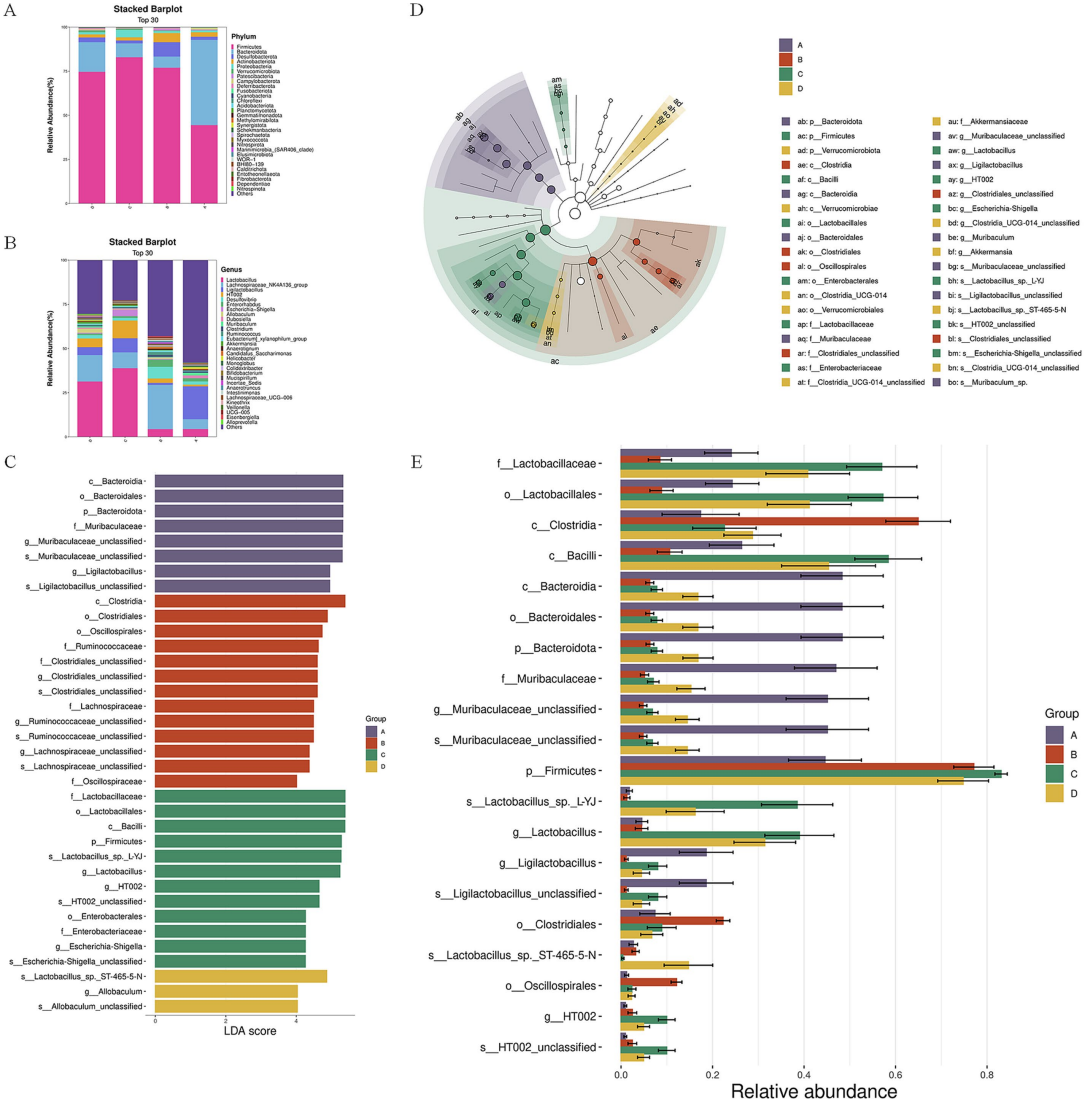
LP25 supernatant modulates gut microbiota composition in DSS-induced ulcerative colitis (UC) mice. **(A)** Relative abundance of gut microbial taxa at the phylum level across all treatment groups, showing restoration of Firmicutes and reduction of Desulfobacterota and Actinobacteriota following LP25 supernatant administration. **(B)** Relative abundance of major genera, illustrating recovery of beneficial bacteria (*Ligilactobacillus*, *Dubosiella*, *Akkermansia*) and reduction of pathogenic genera (*Desulfovibrio*, *Clostridium*, *Anaerotignum*) in a dose-dependent manner. **(C,D)** Linear discriminant analysis effect size (LEfSe) identifying taxa with significantly different abundances among the four groups, with corresponding LDA score distribution (LDA > 3.0, *p* < 0.05). **(E)** Relative abundance of representative differential taxa highlighting microbial restoration in both low-dose and high-dose LP25 supernatant-treated mice. Group labels: **(A)** normal control (NC); **(B)** DSS-induced UC model; **(C)** high-dose LP25 supernatant (H-LP25, 250 mg/kg); **(D)** low-dose LP25 supernatant (L-LP25, 125 mg/kg).

At the phylum level, the control group was dominated by *Firmicutes* and Bacteroidota, typical of a healthy gut microbiota. DSS-induced colitis disrupted this balance, leading to a reduction in *Firmicutes* and an overrepresentation of *Desulfobacterota*, *Actinobacteriota*, and *Patescibacteria.* Treatment with LP25 supernatant, particularly at high dose, reversed these alterations by increasing Firmicutes and reducing pro-inflammatory phyla such as *Desulfobacterota* and *Actinobacteriota* (Figure 4A).

At the genus level, beneficial bacteria including *Ligilactobacillus*, *Dubosiella*, and *Akkermansia* were markedly decreased in DSS-treated mice, whereas opportunistic taxa such as *Desulfovibrio*, *Clostridium*, and *Anaerotignum* were enriched. LP25 supernatant administration normalized these changes in a dose-dependent manner, significantly enriching *Ligilactobacillus* and *Akkermansia* while suppressing pathogenic genera (Figure 4B).

LEfSe analysis (LDA > 3.0, *p* < 0.05) revealed distinct microbial biomarkers among all groups (Figures 4C,D). The control microbiota was characterized by enrichment of *Bacteroidia*, *Muribaculaceae*, and *Ligilactobacillus*, whereas the DSS group exhibited higher abundance of *Clostridia*, and *Oscillospirale*, indicating microbial dysbiosis. In contrast, LP25 supernatant treatment—especially at high dose—promoted enrichment of *Bacilli*, *Lactobacillaceae*, and *Lactobacillus*-related taxa, suggesting partial recovery of the healthy microbial signature.

Analysis of relative abundance of key taxa across all treatments further supported these findings. Both low-dose and high-dose LP25 supernatant groups exhibited significant restoration of *Lactobacillales*, *Lactobacillaceae*, and *Muribaculaceae* compared with the DSS group, with the high-dose group approaching control levels. Notably, the abundance of *Lactobacillus* sp._L-YJ and *Ligilactobacillus* was markedly increased after LP25 treatment, while *Clostridiales* were reduced (Figure 4E).

Collectively, these results demonstrate that LP25 supernatant effectively counteracts DSS-induced dysbiosis by suppressing pathogenic taxa and promoting recolonization of beneficial *Lactobacillus*-associated bacteria, contributing to the restoration of gut microbial homeostasis.

### 3.4 LP25 supernatant more effectively enhances tight junction proteins expression in LPS-induced Caco-2 cells compared with EVs

**Figure 5:**
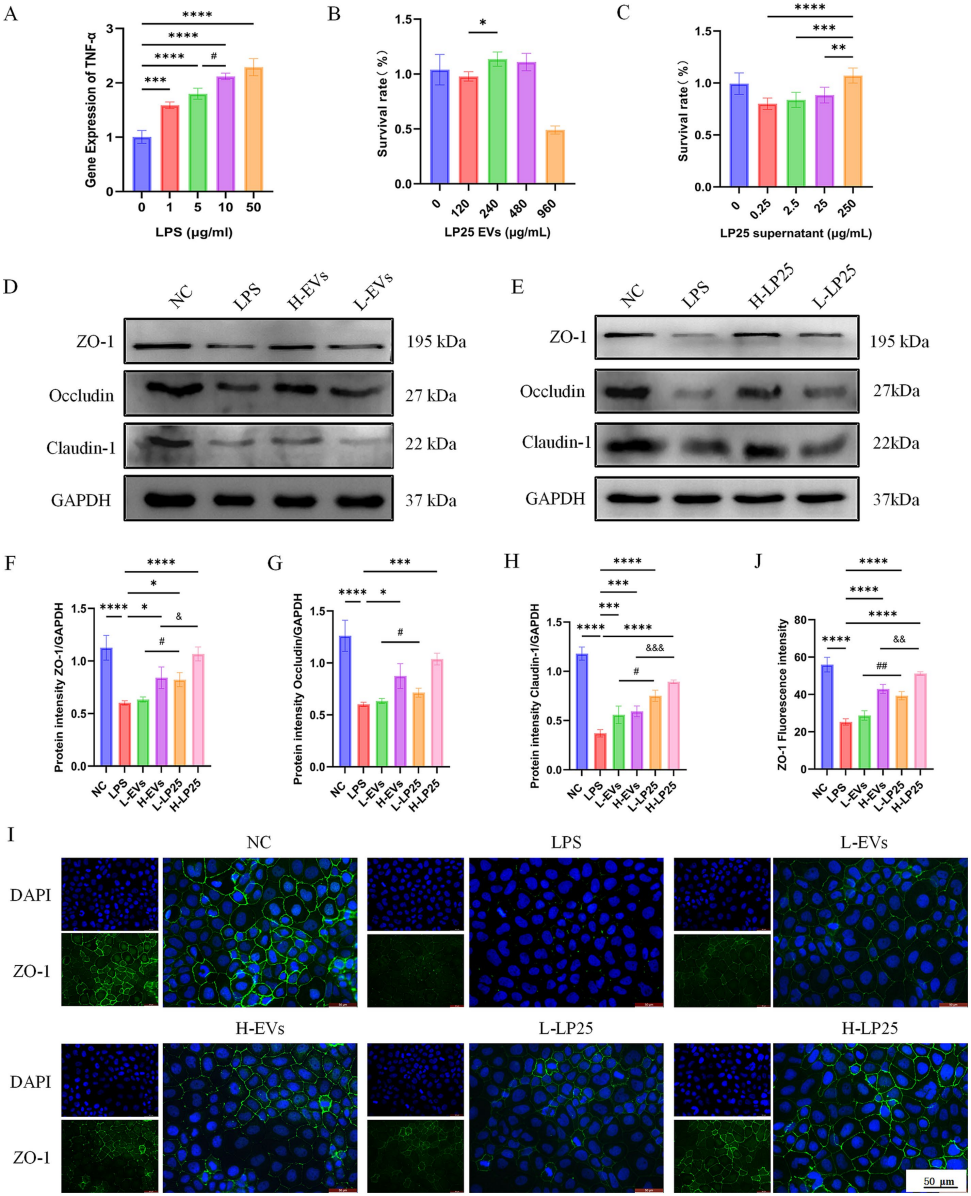
Effects of LP25-derived EVs and LP25 supernatant on TJ proteins expression in LPS-stimulated Caco-2 cells. **(A)** Caco-2 cells were stimulated with lipopolysaccharide (LPS) at concentrations of 0, 1, 5, 10, and 50 μg/mL. TNF-α mRNA expression was measured by qPCR to determine the optimal concentration for inducing inflammation. A concentration of 10 μg/mL was selected for subsequent experiments. ***p < 0.001, ****p < 0.0001 indicate significant differences compared to the 0 g/mL group; ^#^*p* < 0.05 between 5 g/mL and 10 g/mL. **(B)** Cell viability was assessed by CCK-8 assay following treatment with LP25 EVs at concentrations of 120, 240, 480, and 960 g/mL. *p < 0.05 compared to the 120 g/mL group. **(C)** Cell viability was assessed after treatment with LP25 supernatant at concentrations of 0.25, 2.5, 25, and 250 g/mL. Concentrations of 25 g/mL and 250 g/mL were selected for further experiments. **p < 0.01, ***p < 0.001, ****p < 0.0001 vs. 250 g/mL group. **(D,E)** Western blot analysis of TJ proteins (ZO-1, Occludin and Claudin-1) in Caco-2 cells treated with 10 g/mL LPS alone or in combination with low-dose LP25 EVs (120 g/mL), high-dose LP25 EVs (240 g/mL), low-dose LP25 supernatant (25 g/mL), or high-dose LP25 supernatant (250 g/mL). **(F–H)** Densitometric quantification of TJ proteins levels. **(I)** Representative immunofluorescence staining of ZO-1 in each treatment group, with DAPI staining for nuclei (scale bar = 50 m). **(J)** Quantitative analysis of ZO-1 fluorescence intensity. Data are presented as mean ± SD from three independent experiments. *p < 0.05, **p < 0.01, ***p < 0.001, ****p < 0.0001 indicate significant differences compared to the LPS group; ^#^*p* < 0.05, ^##^*p* < 0.01 indicate significant differences between the low-dose LP25 EVs group and the low-dose LP25 supernatant group; ^&^*p* < 0.05, ^&&^*p* < 0.01, ^&&&^*p* < 0.001 indicate significant differences between the high-dose LP25 EVs group and the high-dose LP25 supernatant group.

Given that both LP25 supernatant and EVs improved physiological indicators *in vivo*, we next compared their effects on intestinal epithelial barrier function *in vitro*. Caco-2 cells were stimulated with increasing concentrations of LPS (0, 1, 5, 10, and 50 μg/mL for 24 h), and qPCR analysis of TNF-α mRNA expression revealed the strongest induction at 10 μg/mL, which was subsequently used for all experiments (Figure 5A).

To establish appropriate treatment concentrations, cell viability was assessed using the CCK-8 assay. For EVs, 120 μg/mL and 240 μg/mL were selected as low and high doses, respectively (Figure 5B), while 25 μg/mL and 250 μg/mL were chosen for LP25 supernatant (Figure 5C). These concentrations showed no cytotoxicity to Caco-2 cells.

Western blot analysis demonstrated that both LP25 EVs and supernatant significantly increased the expression of tight junction (TJ) proteins, including ZO-1, claudin-1, and occludin, compared with the LPS-only group. Notably, the upregulation induced by LP25 supernatant was more pronounced than that by EVs (Figures 5D–H). Consistently, immunofluorescence imaging revealed stronger ZO-1 fluorescence signals in supernatant-treated cells than in EV-treated cells, further confirming the superior ability of LP25 supernatant to restore TJ protein expression under inflammatory conditions (Figures 5I,J).

### 3.5 LP25 supernatant preserves intestinal integrity and suppresses TLR4/NF-κB signaling in a Caco-2/RAW 264.7 co-culture model

**Figure 6:**
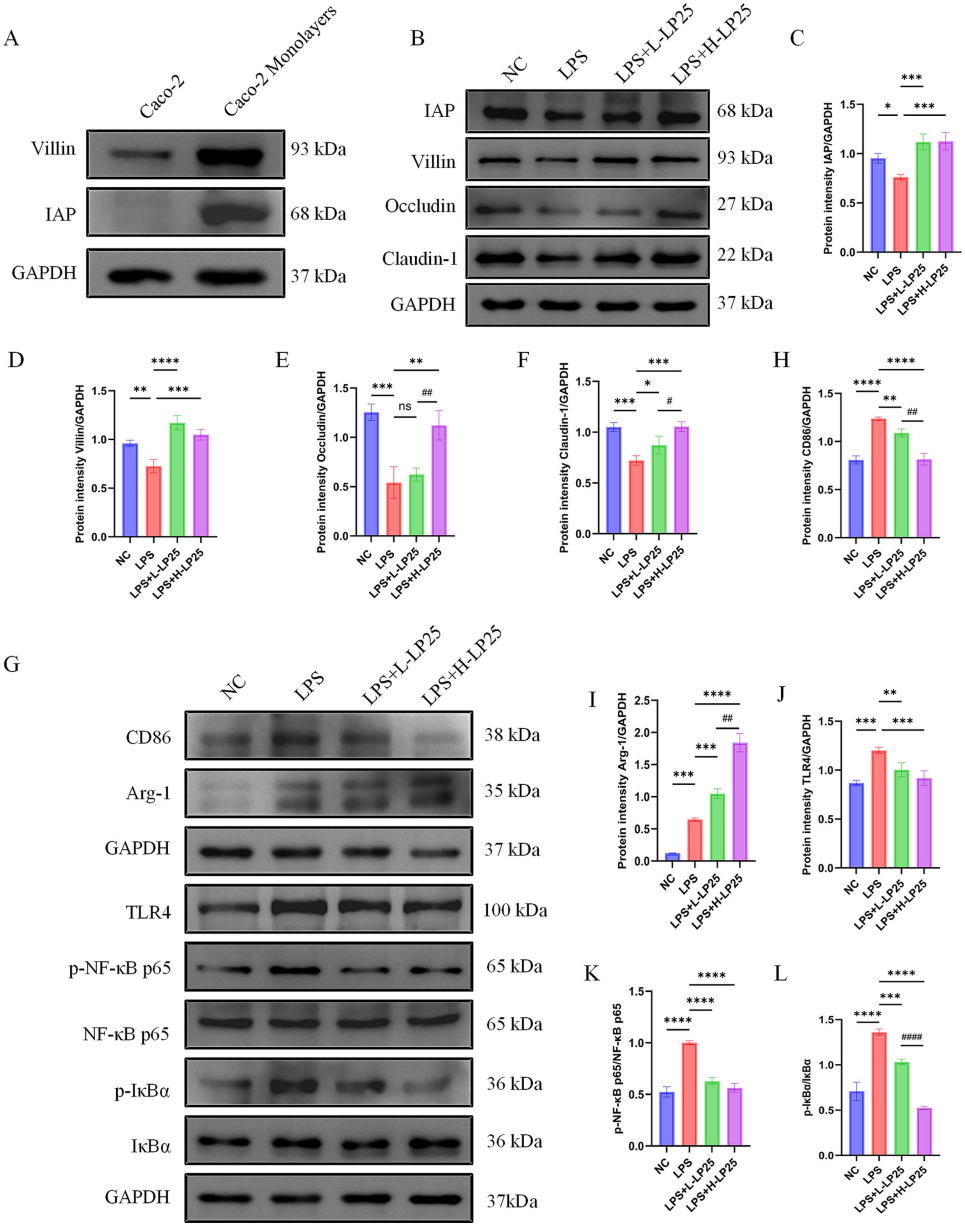
Effects of LP25 supernatant on intestinal barrier integrity and inflammatory response in a Caco-2/RAW 264.7 co-culture model. **(A)** Validation of the Caco-2 monolayer integrity prior to co-culture. **(B)** Western blot analysis of barrier-associated proteins Occludin, Claudin-1, and epithelial differentiation markers IAP and Villin in Caco-2 cells after 18 h of co-culture with RAW 264.7 macrophages under different treatments. **(C–F)** Densitometric analysis of Occludin, Claudin-1, IAP, and Villin proteins expression. **(G)** Western blot detection of CD86, Arg-1, and proteins involved in the TLR4/NF-κB signaling pathway in co-cultured RAW 264.7 macrophages. **(H–L)** Quantitative analysis of proteins expression levels of CD86, Arg-1, TLR4, NF-κB p65, phosphorylated NF-κB p65, IκBα, and phosphorylated IκBα. Data are presented as mean ± SD from three independent experiments. *p < 0.05, **p < 0.01, ***p < 0.001, ****p < 0.0001 indicate significant differences compared to the LPS group; ^#^*p* < 0.05, ^##^*p* < 0.01, ^####^*p* < 0.0001 indicate significant differences compared to the high-dose LP25 supernatant group.

A differentiated Caco-2 monolayer was first established, and the successful epithelial differentiation was verified by Western blot detection of intestinal alkaline phosphatase (IAP) and villin (Figure 6A) (Piana et al., 2008).

In the Caco-2/RAW 264.7 co-culture system, stimulation with LPS (1 μg/mL) markedly disrupted epithelial integrity, as indicated by decreased levels of tight junction proteins. Treatment with LP25 supernatant (L-LP25: 25 μg/mL; H-LP25: 250 μg/mL) significantly restored the expression of Occludin, Claudin-1, IAP, and villin in Caco-2 cells (Figures 6B–F), demonstrating potent barrier-protective effects.

Concurrently, in the basolateral RAW 264.7 macrophages, LP25 supernatant upregulated Arg-1 expression and suppressed CD86 levels, indicating a shift toward an anti-inflammatory M2-like phenotype. Furthermore, LP25 supernatant markedly inhibited activation of the TLR4/NF-κB signaling cascade, as evidenced by reduced phosphorylation of p65 and IκB, along with decreased p-p65/p65 and p-IκB/IκB ratios (Figures 6G–L).

Collectively, these findings suggest that LP25 supernatant maintains intestinal barrier integrity and mitigates inflammation by suppressing TLR4/NF-κB-mediated signaling in the epithelial-macrophage co-culture model.

### 3.6 High-resolution untargeted metabolomics identifies differential metabolites in LP25 supernatant

**Figure 7:**
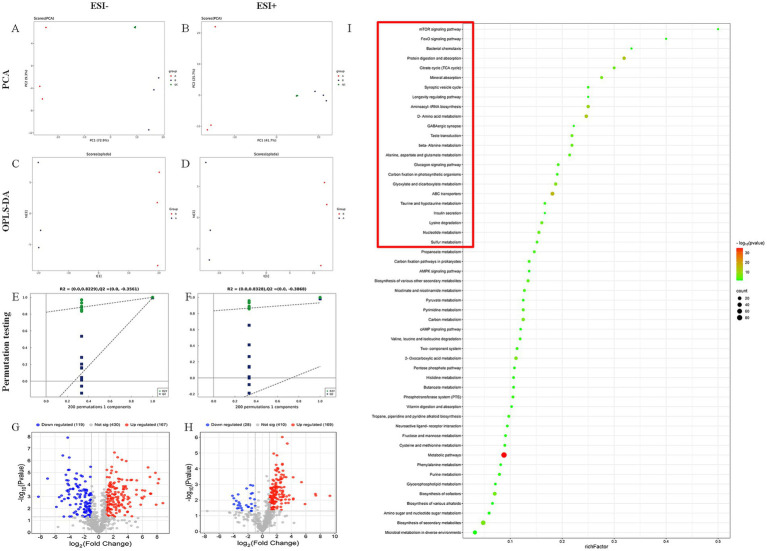
Multivariate statistical analysis of the metabolomic profiles of LP25 supernatant and control samples. **(A,C,E)** Principal component analysis (PCA) score plot, orthogonal partial least squares discriminant analysis (OPLS-DA) score plot, and OPLS-DA permutation test under negative ion mode. **(B,D,F)** Corresponding PCA, OPLS-DA, and permutation plots under positive ion mode. **(G,H)** Volcano plots showing significantly altered metabolites identified under positive and negative ionization modes, respectively. **(I)** KEGG pathway enrichment bubble plot based on significantly different metabolites. Groups: **(A)** concentrated MRS broth control; **(B)** concentrated LP25 supernatant.

Untargeted high-resolution metabolomic profiling was conducted to characterize metabolic differences between LP25 and MRS control samples. Principal component analysis (PCA) demonstrated clear group separation without overlap, indicating distinct metabolic profiles (Figures 7A,B). Consistently, orthogonal partial least squares discriminant analysis (OPLS-DA) further discriminated LP25 from MRS samples along the predictive component (t[1]), with intra-group variation reflected in the orthogonal component (to[1]) (Figures 7C,D). Permutation tests confirmed the robustness of the OPLS-DA model, as both R^2^ and Q^2^ values of randomized models declined progressively with increasing permutation, excluding model overfitting (Figures 7E,F).

Volcano plots revealed the distribution of significantly altered metabolites. In negative ion mode, 167 metabolites were upregulated and 119 downregulated, whereas in positive ion mode, 169 were upregulated and 28 downregulated (Figures 7G,H). Based on fold change ranking, the top 50 differential metabolites from both ion modes (VIP > 1, *p* < 0.05) were mapped to KEGG pathways. Pathways with *p* < 0.05 were visualized using bubble plots, where those with rich factor > 0.15 were considered significant (Figure 7I). Metabolites exhibiting a fold change > 10 within these pathways were further identified as potential bioactive candidates. A total of seven metabolites—D-ribose, histidine, acetylcholine, succinate, glycerophosphate, 5-hydroxylysine, and acetoacetate—were identified as key compounds potentially mediating the effects of LP25 (Supplementary Table S3). Among these, most compounds such as D-ribose, histidine, and succinate are commonly produced by *L. plantarum* strains and participate in core metabolic pathways including carbohydrate and amino acid metabolism. Notably, acetylcholine production has been reported in certain *L. plantarum* strains with neuroactive potential, suggesting that LP25 may share similar biosynthetic capabilities.

## 4 Discussion

**Figure 8:**
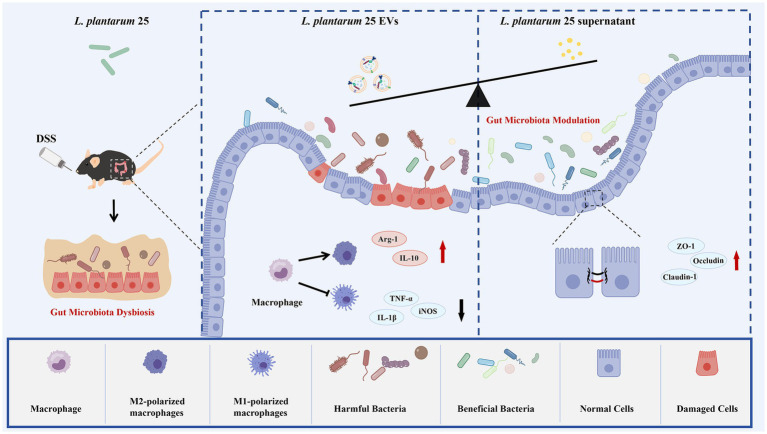
Proposed mechanism of *Lactiplantibacillus plantarum* 25 (LP25)-derived supernatant and extracellular vesicles (EVs) in alleviating acute ulcerative colitis. A DSS-induced mouse model of ulcerative colitis was established to evaluate the therapeutic effects of LP25 supernatant and LP25-derived EVs. Compared with the EV-treated group, mice receiving the LP25 supernatant exhibited more pronounced protective effects, including greater body weight recovery, longer colon length, lower Disease Activity Index (DAI) scores, higher survival rates, and upregulated expression of tight junction proteins. Moreover, LP25 supernatant treatment led to a more balanced gut microbiota profile, characterized by an enrichment of beneficial bacteria and a reduction in pathogenic taxa. *In vitro*, the LP25 supernatant more effectively inhibited LPS-induced inflammatory cytokine production in RAW 264.7 macrophages. In the Caco-2/RAW 264.7 co-culture system, it also suppressed inflammatory signaling and enhanced the expression of epithelial tight junction proteins, collectively contributing to the restoration of intestinal barrier integrity.

Ulcerative colitis (UC) is a chronic inflammatory disease characterized by mucosal barrier disruption, microbial dysbiosis, and immune imbalance (Neurath, 2014). Although current treatments such as 5-aminosalicylic acid and corticosteroids provide symptom relief, they often cause adverse effects and do not restore intestinal homeostasis (Feuerstein et al., 2019; Ungaro et al., 2017). Probiotics have emerged as promising alternatives, and previous studies demonstrated that *Lactiplantibacillus plantarum* ZJ316 alleviated colitis in mice by restoring body weight and downregulating pro-inflammatory cytokines (Gu et al., 2023b). In this study, we identified an anti-inflammatory strain, LP25, and compared the effects of its extracellular vesicles (EVs) and culture supernatant on DSS-induced ulcerative colitis in mice. As shown in Figure 8, LP25 supernatant exerts superior protective effects compared with EVs by enhancing body weight recovery, prolonging colon length, reducing Disease Activity Index (DAI) scores, increasing survival rates, and preserving tight junction protein expression. These effects are further supported by its modulation of gut microbiota composition, enrichment of beneficial bacteria, reduction of pathogenic taxa, inhibition of LPS-induced inflammatory cytokine production in RAW 264.7 macrophages, and enhancement of epithelial barrier integrity in the Caco-2/RAW 264.7 co-culture system, collectively contributing to the restoration of intestinal homeostasis.

A hallmark of UC pathogenesis is the disruption of intestinal barrier function (Bian et al., 2020), largely due to the loss of TJ proteins such as ZO-1, occludin, and claudin-1 (Zhang et al., 2022). Consistent with previous findings that *L. plantarum* JLAU103 can restore barrier function (Liu et al., 2024; Wang J. et al., 2022), both LP25 EVs and supernatant improved DSS-induced symptoms, including body weight loss, colon shortening, and elevated DAI scores. The supernatant, however, showed superior efficacy, significantly enhancing survival and TJ protein expression while reducing α-SMA levels. H&E staining confirmed preservation of epithelial architecture, and *in vitro* experiments demonstrated that LP25 supernatant more effectively restored TJ expression in LPS-stimulated Caco-2 cells. These results suggest that LP25 supernatant exerts stronger barrier-protective activity than its EVs counterpart.

UC is also characterized by intestinal dysbiosis, featuring decreased beneficial bacteria and increased pathogenic taxa (Franzosa et al., 2019; Dong et al., 2022). 16S rDNA sequencing revealed that LP25 supernatant markedly increased the abundance of *Lactobacillus*, *Ligilactobacillus*, *Dubosiella*, and *Akkermansia*, which are SCFA-producing genera supporting epithelial growth and intestinal homeostasis (Liu et al., 2022). This microbial restoration resembles that observed in UC mice treated with Chinese traditional formulations such as Banxia Xiexin Decoction (Luo et al., 2024). Conversely, pathogenic taxa including *Desulfovibrio* and *Clostridium* were significantly reduced. *Desulfovibrio* is generally regarded as a harmful bacterium associated with bacteremia (McDougall et al., 1997), gallstone disease (Hu et al., 2022), obesity (Lin et al., 2022), Parkinson’s disease (Murros et al., 2021), and UC itself (Chen et al., 2021). Members of the genus *Clostridium* (notably *C. difficile*, *C. botulinum*, *C. tetani*, and *C. perfringens*) are linked to a wide range of human and animal diseases (Kiu et al., 2017), with *Clostridioides* difficile infection (CDI) being a major risk factor for both primary and recurrent CDI. Moreover, CDI is known to exacerbate IBD manifestations and increase the risk of complications (Porcari et al., 2023). Thus, LP25 supernatant may alleviate UC partly by reshaping the gut microbiota toward a more beneficial composition.

Immune regulation is another crucial factor in UC pathogenesis, characterized by increased TJ permeability and excessive cytokine production. Co-culture models integrating epithelial (Caco-2) and immune (RAW 264.7) cells effectively mimic *in vivo* epithelial-immune interactions (Wang et al., 2021). In this study, a Caco-2/RAW 264.7 co-culture model was used to assess the effects of LP25 supernatant on barrier integrity and inflammatory signaling, where Caco-2 monolayers represent the intestinal epithelium and LPS-activated RAW 264.7 macrophages simulate intestinal inflammation (Hu et al., 2020; Olejnik et al., 2015). Using this model, we found that LP25 supernatant markedly upregulated tight junction proteins in Caco-2 cells, indicating improved barrier integrity. Meanwhile, in LPS-stimulated macrophages, LP25 supernatant reduced the M1 marker CD86 and increased the M2 marker Arg-1, suggesting a shift toward an anti-inflammatory phenotype. Mechanistically, LP25 supernatant suppressed TLR4/NF-κB signaling by reducing p65 and IκBα phosphorylation, thereby inhibiting NF-κB activation (Prakash et al., 2023; Shao et al., 2023; Liu et al., 2020). These findings indicate that LP25 supernatant protects against UC by enhancing epithelial barrier function and attenuating inflammation through modulation of the TLR4/NF-κB pathway.

To explore the underlying mediators of these protective effects, untargeted metabolomic profiling of concentrated LP25 supernatant was performed, revealing seven metabolites, including acetylcholine and succinate. Acetylcholine, a key neurotransmitter and modulator of intestinal physiology (Middelhoff et al., 2020), is notably depleted in the colonic tissue of IBD patients (Guo et al., 2023). Its detection here is significant given that *Lactiplantibacillus plantarum* can synthesize acetylcholine (Fujita et al., 2024), and similar profiling of *Latilactobacillus sakei* has highlighted acetylcholine as a potential therapeutic metabolite in IBD models (Liu et al., 2023). Another metabolite of interest, succinate, can stimulate IL-25 secretion from tuft cells via SUCNR1, initiating a type-2 immune response that includes ILC2 activation and tuft-goblet cell differentiation (Nadjsombati et al., 2018). IL-25 is a key mediator in mitigating intestinal inflammation along the tuft-ILC2 axis (Hayakawa and Wang, 2018). Exogenous succinate similarly activates SUCNR1 on tuft cells, promoting secretion of IL-25 and IL-13, which drive intestinal stem cell differentiation toward tuft and goblet lineages, thereby supporting epithelial repair and reducing inflammation (Macias-Ceja et al., 2019; Schneider et al., 2018). Collectively, these results suggest that LP25 supernatant alleviates UC by enhancing barrier integrity and modulating inflammation via the TLR4/NF-κB pathway, with acetylcholine and succinate identified as potential mediators. Future work will focus on elucidating the specific role of succinate in DSS-induced colitis and determining the primary metabolite(s) responsible for the observed therapeutic effects.

Given these metabolite-mediated effects, we compared the therapeutic efficacy of LP25-derived extracellular vesicles (EVs) and supernatant in a DSS-induced UC mouse model. The supernatant showed a more pronounced protective effect than EVs. While EVs from beneficial gut bacteria enhance intestinal barrier integrity and reduce gut permeability, primarily through bioactive compounds such as SCFAs, they mainly function as delivery vehicles (Wang et al., 2025). EVs can encapsulate therapeutic molecules for targeted delivery with low immunogenicity, making them suitable for chronic treatments (Liu and Wang, 2023). However, in this acute UC model, their efficacy was limited, possibly reflecting their greater suitability for chronic contexts. Additional challenges include large-scale production (Syromiatnikova et al., 2022) and heterogeneity among EV subtypes, which affect composition, uptake (Liu and Wang, 2023), and biological activity (Yang et al., 2019). In contrast, supernatant offers greater stability, easier acquisition, and consistent bioactivity, making it a more accessible and reliable therapeutic approach.

Although our findings demonstrate that the LP25 supernatant effectively alleviates symptoms in a murine model of acute ulcerative colitis and modulates the gut microbiota, the precise underlying mechanisms remain incompletely understood. Future studies are warranted to identify the key bioactive metabolites responsible for these effects and to elucidate their molecular pathways in greater detail. Nevertheless, therapeutic strategies based on probiotic-derived metabolites offer a promising and innovative direction for the treatment of ulcerative colitis, meriting further investigation.

## 5 Conclusion

In summary, in a DSS-induced colitis mouse model, LP25 supernatant significantly attenuated the disruption of colonic tight junction proteins (ZO-1, occludin, and claudin-1) in UC mice, exerting a protective effect against intestinal injury, and was also associated with improvements in gut microbiota composition. In an *in vitro* co-culture model, LP25 supernatant reduced the expression of macrophage surface marker CD86, increased Arg-1 and TJ proteins expression, and inhibited activation of the TLR4/NF-κB signaling pathway. Collectively, these findings highlight the therapeutic potential of probiotic-derived metabolites as promising candidates for adjunctive treatment of ulcerative colitis.

## Data Availability

The raw data supporting the conclusions of this article will be made available by the authors, without undue reservation.

## Footnotes

1
https://www.bioinformatics.com.cn/
