# Supplementary material for: The supernatant of Lactiplantibacillus plantarum 25 is more effective than extracellular vesicles in alleviating ulcerative colitis and improving intestinal barrier function
Source: Front Microbiol. 2026 Jan 16;16:1742486. doi: 10.3389/fmicb.2025.1742486 (PMC12855493; doi:10.3389/fmicb.2025.1742486)
Supplement: Supplementary file 1 [file Table_1.DOCX]

Supplementary Material

# Supplementary Tables

**Supplementary** **Table 1 Evaluation criteria of DAI ^a^**

| **Score** | **0** | **1** | **2** | **3** | **4** |
| --- | --- | --- | --- | --- | --- |
| Percent body weight loss | 0 | 1-5 | 5-10 | 10-20 | ＞20 |
| Stool consistency | normal | - | mushy | - | diarrhea |
| Rectal bledding | negative | - | positive | - | visible rectal bledding |

a：DAI = (Percent body weight loss + Stool consistency + Rectal bleeding)/3

**Supplementary** **Table 2 Primer sequences for qRT-PCR**

| **Gene** | **Forward sequence (5’-3’)** | **Reverse sequence (5’-3’)** |
| --- | --- | --- |
| *TNF-α*  (Human) | CTCTTCTGCCTGCTGCACTTTG | ATGGGCTACAGGCTTGTCACTC |
| *β-actin*  (Human) | AAGGATTCCTATGTGGGCGAC | CGTACAGGGATAGCACAGCC |

**Supplementary** **Table 3 Metabolites**

| **Metabolite** | **Map Name** | **KEGG** | **HMDB** |
| --- | --- | --- | --- |
| D-Ribose | Bacterial chemotaxis | C00121 | HMDB0000283\| |

**Supplementary** **Table 3 Metabolites (continued)**

| **Metabolite** | **Map Name** | **KEGG** | **HMDB** |
| --- | --- | --- | --- |
| Histidine | Protein digestion and absorption | C00135 | HMDB0000177 |
| Acetylcholine | Synaptic vesicle cycle | C01996 | HMDB0000895 |
| Succinate | GABAergic synapse | C00042 | HMDB0000254 |
| Glycerophosphate(2) | ABC transporters | C00093 | HMDB0000126 |
| 5-hydroxylysine | Lysine degradation | C16741 | HMDB0000450 |
| Acetoacetic acid | Lysine degradation | C00164 | HMDB0000060 |
